# Characterization of Pyroptosis-Related Subtypes *via* RNA-Seq and ScRNA-Seq to Predict Chemo-Immunotherapy Response in Triple-Negative Breast Cancer

**DOI:** 10.3389/fgene.2022.788670

**Published:** 2022-03-21

**Authors:** Chenlu Li, Jingjing Pan, Yinyan Jiang, Yanzhi Wu, Zhenlin Jin, Xupeng Chen

**Affiliations:** ^1^ Department of Gastroenterology, Affiliated Yueqing Hospital, Wenzhou Medical University, Wenzhou, China; ^2^ Department of Laboratory Medicine, The First Affiliated Hospital of Wenzhou Medical University, Wenzhou, China; ^3^ Department of Hematopathology, The First Affiliated Hospital of Wenzhou Medical University, Wenzhou, China; ^4^ Department of Rheumatology, The First Affiliated Hospital of Wenzhou Medical University, Wenzhou, China

**Keywords:** pyroptosis, triple-negative breast cancer, molecular subtype, tumor microenvironment, prognosis, single-cell RNA sequencing

## Abstract

Triple-negative breast cancer (TNBC) is associated with poor prognosis and invalid therapeutical response to immunotherapy due to biological heterogeneity. There is an urgent need to screen for reliable indices, especially immunotherapy-associated biomarkers that can predict patient outcomes. Pyroptosis, as an inflammation-induced type of programmed cell death, is shown to create a tumor-suppressive environment and improve the chemotherapeutic response in multiple tumors. However, the specific therapeutic effect of pyroptosis in TNBC remains unclear. In this study, we present a consensus clustering by pyroptosis-related signatures of 119 patients with TNBC into two subtypes (clusterA and clusterB) with distinct immunological and prognostic characteristics. First, clusterB, associated with better outcomes, was characterized by a significantly higher pyroptosis-related signature expression, tumor microenvironment prognostic score, and upregulation of immunotherapy checkpoints. A total of 262 differentially expressed genes between the subtypes were further identified and the Ps-score was built using LASSO and COX regression analyses. The external GEO data set demonstrated that cohorts with low Ps-scores consistently had higher expression of pyroptosis-related signatures, immunocyte infiltration levels, and better prognosis. In addition, external immunotherapy and chemotherapy cohorts validated that patients with lower Ps-scores exhibited significant therapeutic response and clinical benefit. Combined with other clinical characteristics, we successfully constructed a nomogram to effectively predict the survival rate of patients with TNBC. Finally, using the scRNA-seq data sets, we validated the landscape of cellular subtypes of TNBC and successfully constructed an miRNA-Ps-score gene interaction network. These findings indicated that the systematic assessment of tumor pyroptosis and identification of Ps-scores has potential clinical implications and facilitates tailoring optimal immunotherapeutic strategies for TNBC.

## Introduction

As the most common tumor with high morbidity and mortality in women, breast cancer (BC) has a poor prognosis and exacerbates a critical social burden worldwide (Global Burden of Disease Cancer Collaboration et al., 2017). Triple-negative breast cancer (TNBC), accounting for 10%–17% among all BCs, is a special subtype characterized by negative human epidermal growth factor receptor 2 (HER2), progesterone receptor (PR), and estrogen receptor (ER) (Lin et al., 2012). Due to the absence of the corresponding receptors, patients with TNBC fail to benefit from endocrine targeted therapy and HER2-targeted agents; hence, chemotherapy and surgery remain the most common treatment for patients with TNBC (Bergin and Loi, 2019). Recently, the rapid rise of immunotherapy with the combination of cisplatin or other platinum drugs, including anti-programmed cell death (PD)-1 and PD-ligand 1 (PD-L1) agents, has brought a new therapeutic landscape for patients with TNBC who did not benefit from conventional chemotherapy, radiation, or surgery (Nolan et al., 2017). However, in clinical practice, some patients with TNBC are still lacking an effective therapeutical response to immunotherapy due to genetic and biological heterogeneity (Vikas et al., 2018). Therefore, it is crucial to identify novel subtypes and screen reliable biomarkers (especially immunotherapy-related biomarkers) that can predict outcomes of patients with TNBC.

In the clinical setting, the TNM stage system is acknowledged as the most frequently used tool to predict the prognosis of patients with TNBC, which majorly depends on the inherent anatomical abnormity, including tumor size, lymph node situation, and distant metastatic status (Park et al., 2019). However, the occurrence of biological and tumor genetic heterogeneity makes it challenging for the TNM system to predict disease progression and prognosis (Park et al., 2019). Pyroptosis is a form of pro-inflammatory programmed cell death (PCD) that cleaves the gasdermin D (GSDMD) protein by classical or nonclassical pathways and triggers the production and release of cytokines (including inactive cytokines like IL-18 and IL-1β) to induce a strong inflammatory response (Yang et al., 2016). Pyroptosis is reported to create a tumor-suppressive environment by releasing inflammatory factors; therefore, inducing pyroptosis in tumors via chemotherapeutic drugs could produce antitumor effects (Shi et al., 2015). *In vitro*, Nathalia et al. demonstrate that omega-3 fatty acids can induce pyroptosis in TNBC cells via inducing the active CASP1 increase, further leading to the cleavage of GSDMD, formation of membrane pores, and the release of IL-1β (Pizato et al., 2018). However, the exact contribution of pyroptosis on the therapeutical response of immunotherapies and its role in the prognosis of TNBC remains unclear.

The classification of patients with TNBC based on transcriptome profiles via next-generation sequencing is considered a novel method to quickly indicate biological characteristics and help screen for the most appropriate treatment strategies (He et al., 2018). Besides conventional expression profiles, various biological signatures are also applied to identify novel molecular subtypes for the prognosis of TNBC, such as autophagy-related signatures (Kim et al., 2012), N6-methyladenosine (Wu et al., 2021), immune cell infiltration (Harano et al., 2018), etc. In this study, we aimed to build a novel scoring model (called Ps-scores) based on pyroptosis-related signatures to identify clustering subtypes of TNBC and correlate the characteristics of each subtype with prognosis, immunotherapy, and immune cell infiltration in patients with TNBC. Combining the Ps-scores and other classical clinical features, the predicted model was established to improve prognostic risk stratification and facilitate the decision making of treatments for patients with TNBC. Moreover, using single-cell RNA sequencing (scRNA-seq) technology, we successfully validated the potential cellular subtypes of TNBC and expounded the predominant expression characteristics of Ps-score-related genes in each cluster. Finally, the targeted miRNAs were predicted by combining multiple databases with differentially expressed miRNAs (DEmiRNAs), whereas the miRNA-Ps-score signature interaction network was further constructed to visualize the potential regulatory relationship. These results imply the potential links among the pyroptosis-related scores, immune microenvironment, prognosis, and response to immunotherapy for patients with TNBC. Our findings provide new insight into the prognostic signatures of TNBC and will help develop promising strategies for TNBC immunotherapy.

## Materials and Methods

### TNBC Data set Preparation and Preprocessing

Transcriptome profiling data (FPKM value) of 1217 BC samples with their corresponding clinical data were downloaded from The Cancer Genome Atlas (TCGA) data sets (https://portal.gdc.cancer.gov/). Through screening the “negative HER2, PR, and ER” status based on clinical data, we finally identified 119 patients with TNBC and comprehensive clinical information. Other microarray data sets of 819 patients with TNBC and prognostic information were also downloaded from the Gene Expression Omnibus (GEO) data sets (https://www.ncbi.nlm.nih.gov/geo/), including 120 ER-negative BC in GSE16446, 198 HER2-negative BC in GSE25065, 310 HER2-negative BC in GSE25055, 107 TNBC in GSE58812, and 84 TNBC in GSE157284. In addition, corresponding miRNA sequencing data sets and mutation files were also obtained from the TCGA-BRCA to investigate the miRNA regulatory mechanism, and transcriptome profiling of 179 normal breast tissues was obtained from the Genotype-Tissue Expression (GTEx) database as normal controls (Carithers and Moore, 2015). The “ComBat” algorithm of the “sva” package was further applied to remove the nonbiological technical biases due to batch effects between different data sets (Leek et al., 2012). To remove the false positives caused by batch effects, we selected several stable internal reference genes (*HPRT1*, *PPIA*, *RPS13*, *TBP*, *GAPDH*, and *HMBS*) to perform the PCA analysis, which are reported as valid reference genes for human BC cell lines by Liu et al. (2015). Moreover, the IMvigor210 data sets (Hoffman-Censits et al., 2016), including 316 metastatic urothelial carcinomas (mUCs) with immunotherapy, were applied to investigate the therapeutic reaction, and the scRNA-seq data of 1534 cells from six patients with TNBC (GSE 118389) were used to validate molecular subtypes for TNBC. Detailed information on these data sets is listed in Supplementary Table S1.

### Identification of Pyroptosis-Related Signatures

According to previous studies, the caspase family, especially caspase1/4/5/8 (CASP1/4/5/8) was reported to specifically cleave GSDMD to further activate pyroptosis (Shi et al., 2015; Orning et al., 2018). In addition, Zhang et al. (2020), also found that CASP3 and granzyme B (GZMB) could convert cell apoptosis into pyroptosis through cleaving gasdermin E (GSDME). Granzyme A (GZMA) was also considered to be essential in inducing cell pyroptosis by cleaving gasdermin B (GSDMB) (Zhou et al., 2020). Moreover, inflammasome-associated families, such as absent in melanoma 2 (AIM2) and nucleotide-binding domain and leucine-rich repeat receptor (NLR), are demonstrated to induce the pyroptosis process through activating CASP1 and the release of IL1β and IL18 (Man and Kanneganti, 2015; Karki and Kanneganti, 2019). Therefore, based on the published research, a total of 33 pyroptosis-related signatures were chosen, and 24 genes were retained for subsequent analysis after filtering out the signatures with low expression (sum FPKM value of all samples less than one).

### Consensus Cluster Analysis for Pyroptosis-Related Signatures in TNBC

Based on the expression of pyroptosis-related signatures, we performed hierarchical clustering analysis and applied the “ConsensusClusterPlus” R package (Wilkerson and Hayes, 2010) to conduct unsupervised clustering based on Euclidean distance and Ward’s linkage methods 1000 repeated times to ensure the classification stability. During the process, the clusters from 2 to 9 were performed, respectively, and the optimal clustering model was determined based on the consensus cumulative distribution function (CDF) plot. Moreover, we performed multiple comparisons among different pyroptosis-subtypes, including for the tumor microenvironment (TME), prognosis, and vital clinical-pathological phenotypes to explore their characteristics. The R packages “survival” (Therneau and Lumley, 2015) and “survminer” (Kassambara et al., 2017) were used to perform Kaplan–Meier survival analysis and draw survival curves between pyroptosis subtypes.

### Identification of Differentially Expressed Genes (DEGs) and Functional Enrichment Analysis

To identify the DEGs between pyroptosis subtypes, the empirical Bayesian algorithm was applied through the “Limma” R package (Smyth, 2005), and the significance cutoff was set as adjusted *p* < .05 and absolute fold-change >1. To clarify the biological function and characteristics of pyroptosis clusters, Kyoto Encyclopedia of Genes and Genomes (KEGG) enrichment analysis was performed by using the “ClusterProfiler” R package (Yu et al., 2012), and the results were visualized using the “ClueGO” plugin in Cytoscape v3.7.1 (Bindea et al., 2009).

### TME Cell Infiltration and Gene Set Variation Analysis

To evaluate the immune cell infiltration (ICI) characteristics of TNBC tissues, we used the “CIBERSORT” R package (Chen et al., 2018) to quantitatively analyze the infiltration levels of different immune cells with the LM22 signatures by 1000 random permutations. The tumor purity scores, ICI levels, and stromal contents in different samples were evaluated via the “ESTIMATE” algorithm (Yoshihara et al., 2013). Moreover, through the “c2. cp.kegg.v6.2. symbols” data sets downloaded from the MSigDB database, we performed GSVA using the “GSVA” R package and drew a heatmap to exhibit the different immunogenic pathways (Hanzelmann et al., 2013).

### Definition of Immune Characteristics Between High and Low Ps-Score Groups

To further identify a novel index representing the characteristics of the pyroptosis subtypes, we conducted univariate Cox proportional hazards regression analysis for overall survival (OS) to preliminarily screen significant genes through using the “coxph” function in the “survival” R package. Subsequently, to remove the multicollinearity among these candidate genes, LASSO regression was applied to screen independent prognosis-related genes with the optimal penalty parameter and a minimum 10-fold cross-validation (Ranstam and Cook, 2018). After further adjustment, the multivariate Cox regression (stepwise model) was conducted to identify hub genes, and the coefficients obtained from the regression algorithm were used to acquire the Ps-score based on the following formula: Ps_score=val(Gene1)×β1+val(Gene2)×β2+…+val(Gene n)×βn . The val (Gene) represents the expression FPKM value of each gene and β the corresponding regression coefficient. Moreover, according to the above formula, the Ps-scores of patients with TNBC were separately calculated, and the patients were divided into high and low subgroups according to the median value as the cutoff value (Sullivan et al., 2004). We also made similar comparisons between high and low Ps-score groups, including the TME, ICI, clinical phenotypes, pyroptosis-related signatures, and correlation of GSVA pathways based on the other four GEO data sets described earlier.

### Construction and Evaluation of the Pyroptosis-Related Prediction Model

The multivariate Cox regression (stepwise model) was applied to construct the prognostic model for TNBC-combined Ps-scores and other clinical features, including age, clustering subtypes, clinical stages, and TNM stages. Variables with *p*-values < .05 were included in the Cox regression model, and the nomogram was further constructed to predict the probability of 1-, 3-, and 5-year survival in patients with TNBC using the “survival” R package. To evaluate and validate the prediction capability of the nomogram, we calculated the concordance index and plotted the calibration curves for 3- and 5-year survival through a bootstrapping method with 1000 resamples. To further investigate the expression of the Ps-score-related genes at the protein level, the Human Protein Atlas (HPA) (Ponten et al., 2008) was used to display the results of the immunohistochemistry (IHC) technique. The detailed information of patients is included in Supplementary Table S17.

### Exploration of the Significance of Ps-Scores in Response to Clinical Immunotherapy

Based on the IMvigor210 data sets with atezolizumab treatment, we performed a comprehensive comparison between different Ps-score subgroups, including response to immunotherapy, immune phenotype, and clinical remission rate. Moreover, to evaluate the potential therapeutic value of Ps-scores in chemotherapy for TNBC, we calculated the half-maximal inhibitory concentration (IC_50_) of common chemotherapeutic drugs based on the Genomics of Drug Sensitivity in Cancer (GDSC) databases (Yang et al., 2013). Antitumor drugs such as 5-fluorouracil, cisplatin, docetaxel, doxorubicin, and paclitaxel are recommended for BC treatment by current clinical guidelines. Differences in IC_50_ of these chemotherapeutic drugs between Ps-score subgroups were compared by Wilcoxon test with the results exhibited in box diagrams using the “ggpubr” R package (Whitehead et al., 2019).

### Validation of Molecular Subtypes Based on scRNA-Seq Analysis

To validate molecular clusters and further seek biomarkers of each cluster, the Seurat pipeline was selected for subsequent analysis. Using the Seurat package v3.0 (Butler et al., 2018), we transformed the data matrix into a “Seurat object” through the “CreateSeuratObject” R function and performed the necessary quality control. The violin diagram exhibited the number of sequencing reads per sample and the expression of mitochondrial genes (Supplementary Figure S2A). Further, to remove the influence from mitochondrial and extreme genes, we kept the number of sequenced genes at 200–10,000, directly including the majority genes, and removed the cells with average gene expression <10 and mitochondrial genes >5%. Then, we conducted data standardization through the “NormalizeData” function with the method of “LogNormalize” and used the top 1500 variable counts to perform PCA using the “RunPCA” function (Supplementary Figures S2B,C). Subsequently, *t*-distributed statistical neighbor embedding (tSNE) was applied to visualize the density clustering, and the “SingleR” package was applied for cell-subtype annotation based on the marker genes (Aran et al., 2019). Moreover, the pseudotime trajectory analysis was further performed using the “Monocle” package v2.0 to expound the potential inner relationship among these cell clusters (Trapnell et al., 2014).

### Prediction of Potential miRNA Targets for Prognosis-Associated Signatures

The miRNAs targeting Ps-score-related genes were predicted based on the following databases: TargetScan (http://www.targetscan.org/), starBase (http://starbase.sysu.edu.cn/starbase2/index.php), miRTar (https://mirtarbase.cuhk.edu.cn/), and miRDB (http://www.mirdb.org/). The expression of TNBC miRNA was downloaded from the TCGA-BRCA data sets, and the DEmiRNAs were further identified by the “Limma” R package. Subsequently, we identified the intersection of predicted miRNA by four databases and DEmiRNAs as regulated miRNAs for each hub gene and further visualized the miRNA–mRNA interaction network using Cytoscape v3.7.1.

## Results

### Overview of Genetic and Biological Characteristics of Pyroptosis-Related Signatures in TNBC

**FIGURE 1:**
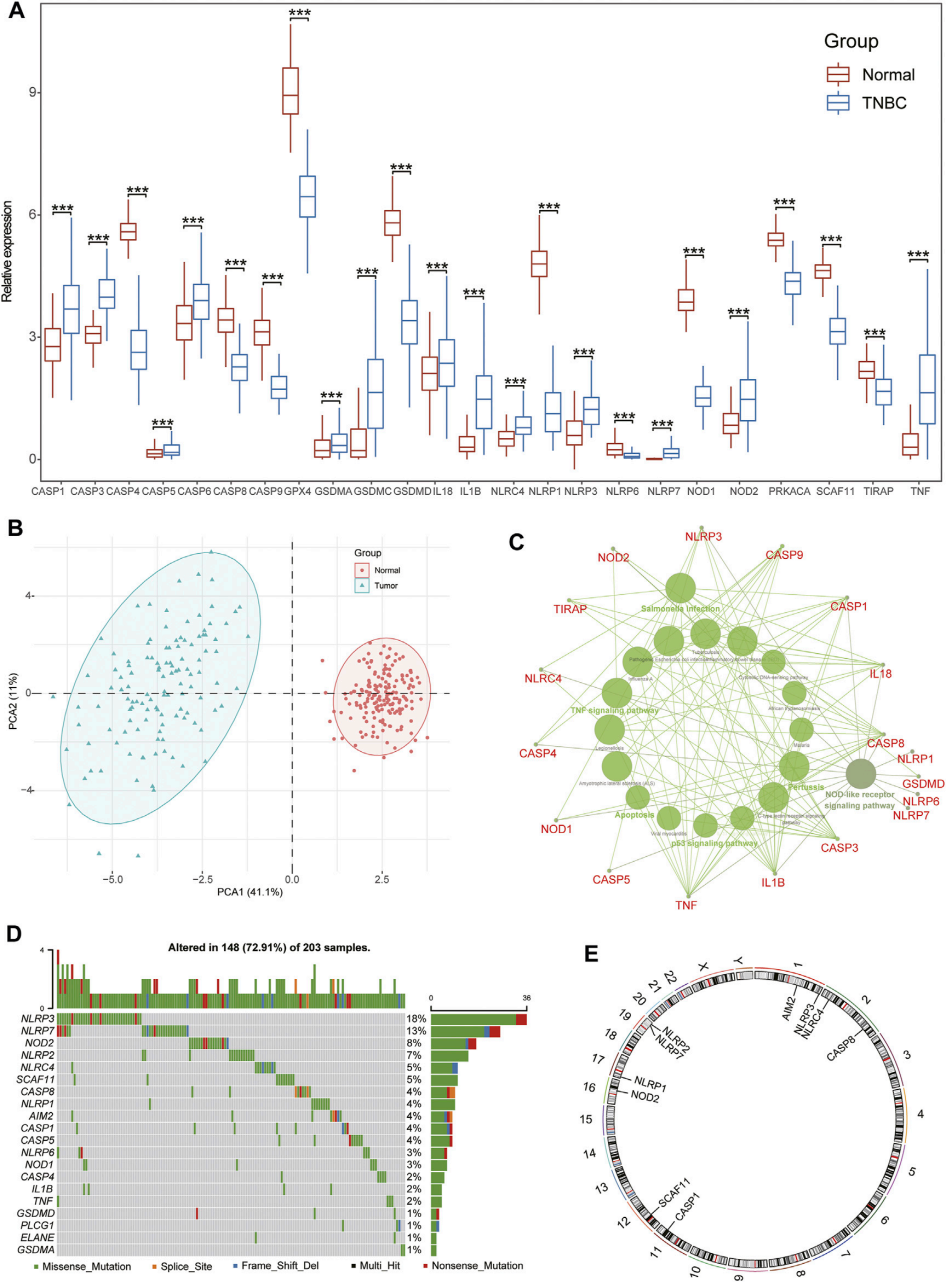
Characteristics of pyroptosis-related signatures in patients with TNBC. **(A)** The expression of pyroptosis-related signatures between normal tissues and TNBC tissues from GTEx and TCGA data sets; Wilcox test, *p < .05, **p < .01, ***p < .001; ns, not statistically significant. **(B)** PCA showing pyroptosis-related signatures sorted TNBC and control tissues into two clusters. **(C)** The KEGG functional analysis of pyroptosis-related signatures. **(D)** The landscape of mutation profiles in patients with breast cancer from TCGA-BRCA cohort. **(E)** The location of the top 10 pyroptosis-related signatures with the most frequent mutations on the 24 human chromosomes.

After a series of rigorous screening and quality control steps, a total of 24 pyroptosis-related signatures remained for subsequent analysis in our study (Supplementary Table S2). Combined with the normal tissues in GTEx data sets, we first compared the expression of pyroptosis-related signatures between patients with TNBC and normal controls. We found that most pyroptosis-related genes were significantly upregulated in TNBC groups, including *CASP1/3/5/8*, *GSDMA/C*, *NLRC4/P3/P7*, *IL18*, *IL1β*, and *TNF* (Figure 1A). PCA indicated that the expression of these pyroptosis-related signatures could be used to divide the TNBC samples and controls into two distinct clusters (Figure 1B). In addition, the PCA of internal reference genes revealed that nonsignature genes failed to discriminate TNBC and control cohorts, indicating that the separation created a true distinction based on the pyroptosis-related DEGs in patients with TNBC (Supplementary Figure S1A). Moreover, the KEGG functional enrichment analysis revealed that these pyroptosis-related genes were predominantly focused on infectious diseases, immune response, and cellular signal conditioning mechanisms, including the NLR signaling pathway, p53 signaling pathway, TNF signaling pathway, and apoptosis (Figure 1C, Supplementary Table S3). In terms of genetics, 148 of the 203 samples (72.91%) manifested pyroptosis-related signatures in mutations and the NLR families, especially NLRP3 and NLRP7, exhibiting the highest frequency of mutations (Figure 1D, Supplementary Table S4). Moreover, the top 10 pyroptosis-related genes with the most frequent mutations were located on the 24 human chromosomes (Figure 1E).

### Identification of a TNBC Cluster Pattern Based on Pyroptosis-Related Signatures

**FIGURE 2:**
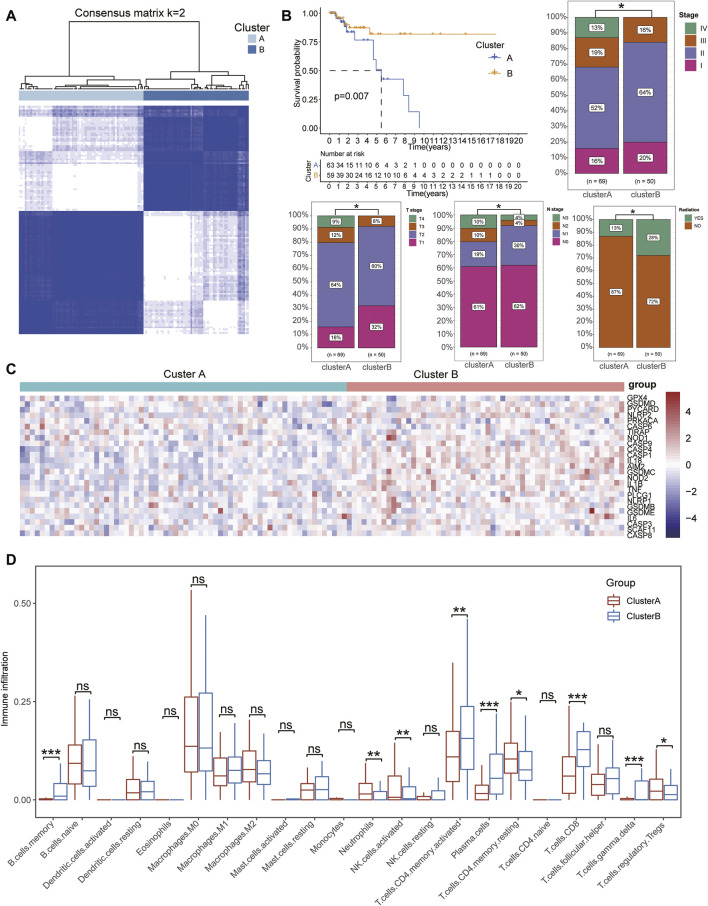
Identification of pyroptosis-related subtypes in TNBC. **(A)** Consensus clustering matrix for *k* = 2 in patients with TNBC. **(B)** Kaplan–Meier curves of OS for patients with TNBC divided into two subtypes. ClusterB was negatively associated with severe clinical stages, including pathological and TMN stages. **(C)** Heatmap showing the expression of pyroptosis-related signatures upregulated in ClusterB subtypes. **(D)** Boxplots show the difference in immune cell infiltration between ClusterA and ClusterB. Wilcox test, *p < .05, **p < .01, ***p < .001.

**FIGURE 3:**
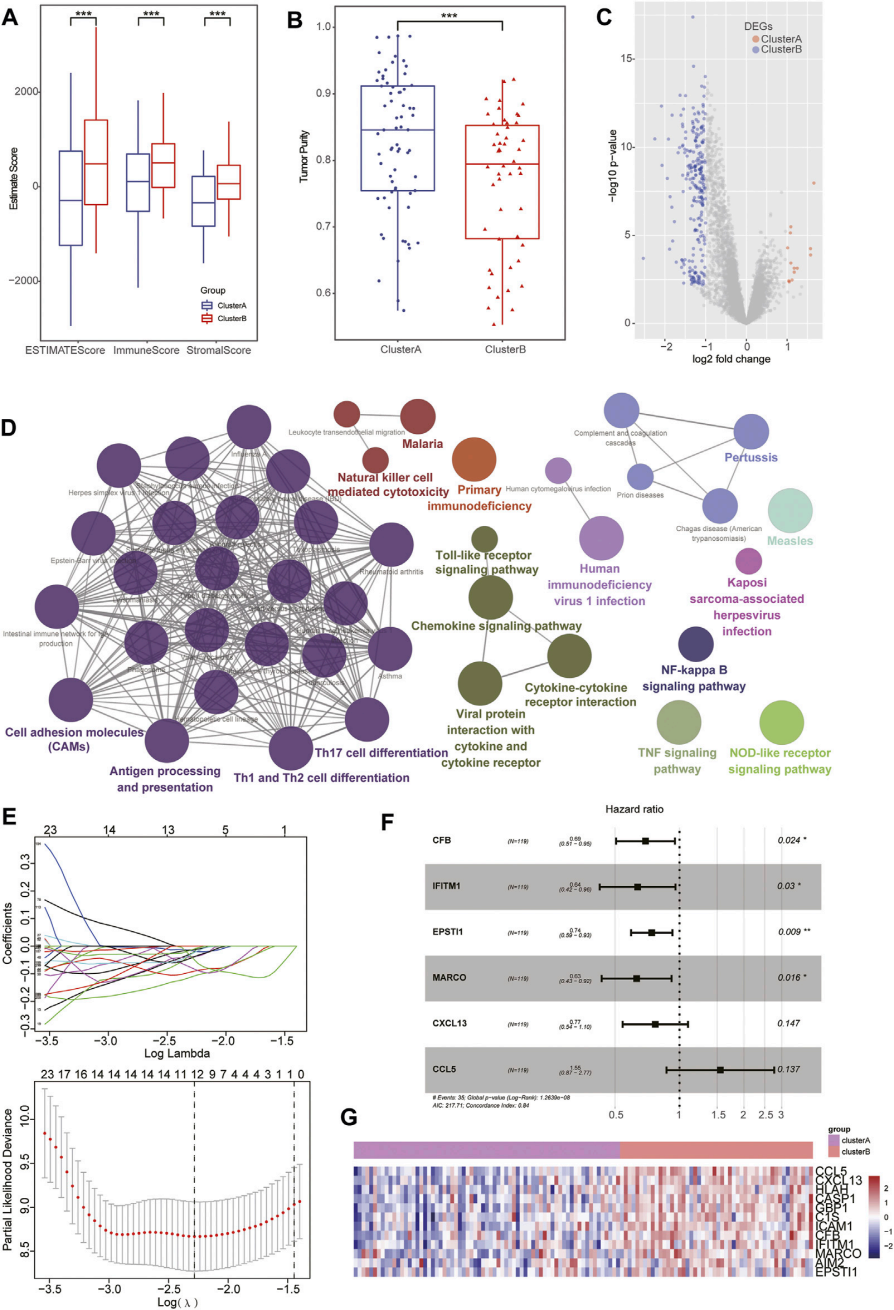
Identification of DEGs based on pyroptosis-related clusters. **(A–B)** Box plot showing higher stromal and immune scores with lower tumor purity detected in patients of clusterB than the clusterA group. **(C)** Volcano plots displaying the up- and downregulated DEGs between two subgroups in TNBC cohorts. **(D)** Bubble diagram showing the results of KEGG enrichment analysis of the subtypes. **(E)** LASSO coefficient profiles of 12 prognostic related genes and 10-times cross-validation for tuning parameter selection in the LASSO model. **(F)** Forest map displaying the HR and *p*-value of six hub genes after multivariate Cox regression analysis. **(G)** Heatmap showing the distinct expression of six hub genes between pyroptosis-related clusters.

Based on the expression of pyroptosis-related signatures, we used an unsupervised clustering method to identify the subtypes of patients with TNBC and identified *k* = 2 as the optimum clustering model from *k* = 2 to *k* = 9 clustering with the least area under the consensus CDF curve for 69 patients in clusterA and 50 patients in clusterB (Figure 2A, Supplementary Table S4; Supplementary Figure S1B). To further clarify the intrapatient heterogeneity of patients with TNBC, we performed a comparison in the clinical differences between subtypes and found that patients in clusterB were negatively associated with severe clinical stages, including the pathological and TMN stages. Furthermore, the survival analysis showed that patients in clusterB had a longer median survival time than those in clusterA with more patients in clusterB also receiving radioactive treatments, indicating that patients with TNBC in clusterB might have a better prognosis (Figure 2B). There was no significant difference in other clinical indexes including age, sex, M stage, and the ratio of pharmaceutical and surgical therapies (Supplementary Figure S1C). Notably, the expression of pyroptosis-related gene signatures was significantly increased in patients in clusterB compared with that of the clusterA cohort (Figure 2C). In terms of the immune infiltration scores, adaptive immune response-related lymphocytes (including memory B cells, activated memory CD4^+^ T cells, plasma cells, CD8^+^ T cells, and gamma delta T cells) were significantly increased in patients in clusterB compared with the clusterA cohort. However, innate immunity and immunoregulation-related cells were significantly infiltrated in clusterA cohorts, including neutrophils, activated natural killer cells, resting memory CD4^+^ T cells, and regulatory T cells (Tregs) (Figure 2D, Supplementary Table S5). Higher stromal scores and immune scores with lower tumor purity were also detected in patients in clusterB compared with the clusterA groups (Figures 3A,B).

### Identification of DEGs Based on Pyroptosis-Related Clusters

Considering the biological characteristics of immune subtypes in TNBC, we conducted a DEG analysis between the two subtypes. Through comparing clusterA with clusterB groups, a total of 262 DEGs (including 13 clusterA- and 249 clusterB-related genes) in TNBC were identified (Figure 3C, Supplementary Table S6). To further interpret biological processes and pathways of pyroptosis-related subtypes, these DEGs were chosen to perform KEGG functional analysis. The results showed that clusterA-related genes were not enriched in any significant pathways while the clusterB-related signatures were predominantly enriched in immune activation-associated pathways, including natural killer cell–mediated cytotoxicity, the toll-like receptor signaling pathway, chemokine signaling pathway, cytokine–cytokine receptor interaction, NF-kappa B signaling pathway, TNF signaling pathway, Th17 cell differentiation, and Th1 and Th2 cell differentiation (Figure 3D, Supplementary Table S7).

### Development of Ps-Score and Characteristic of Ps-score-related Subgroups

**FIGURE 4:**
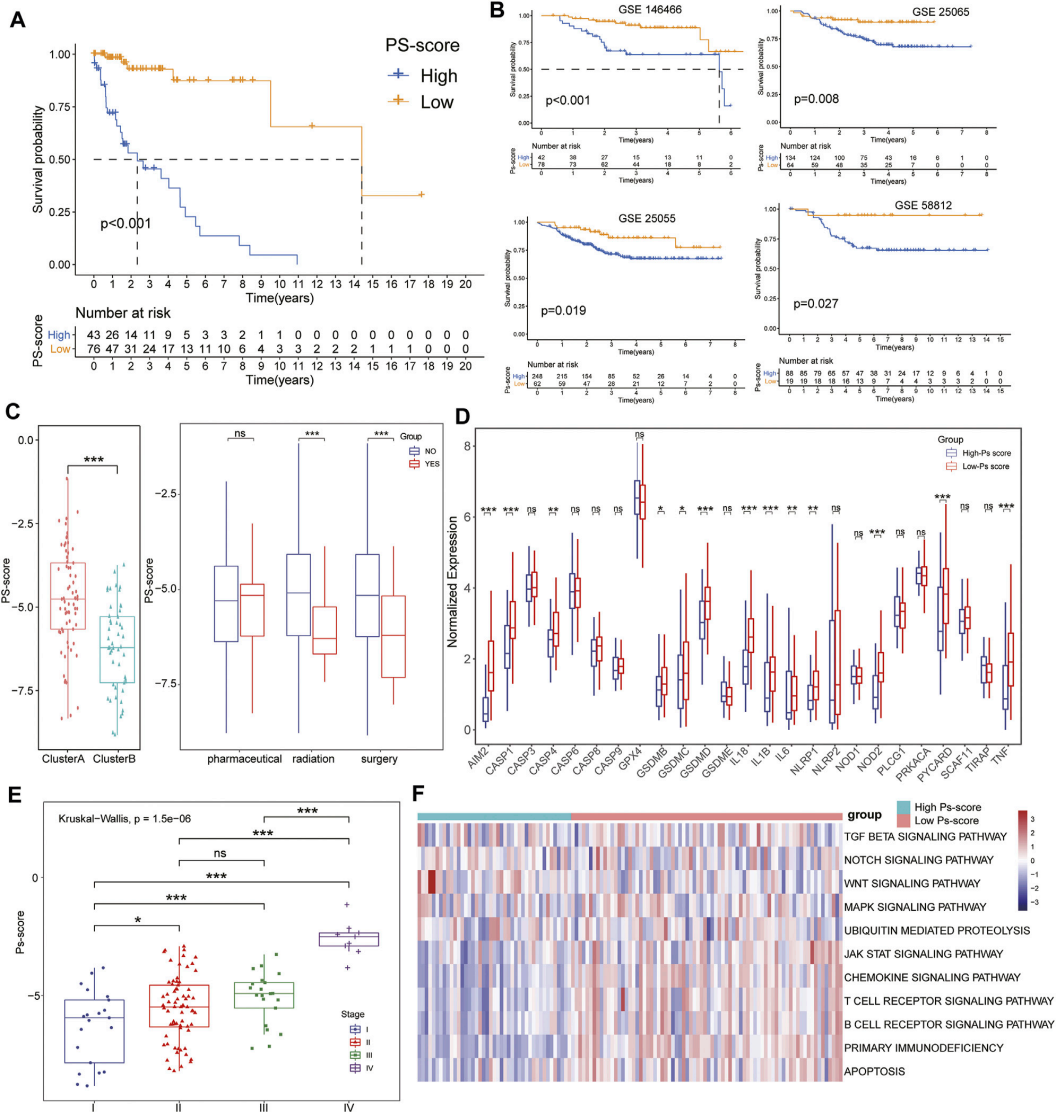
Characteristics of Ps-scores and correlation to pyroptosis in TNBC. **(A–B)** Kaplan–Meier curves of overall survival (OS) for the patients with TNBC in the high- and low-Ps-score groups in the TCGA **(A)** or GEO data sets **(B)**. **(C, E)** The box plots show that ClusterB possessed a lower level of Ps-scores associated with pharmaceutical and surgical therapies, and lower pathological stages. **(D)** Box diagram displaying the expression of pyroptosis-related signatures significantly increased in the low Ps-score groups. **(F)** Heatmap used to visualize pathways analyzed by GSVA showing the active biological pathways in distinct pyroptosis-related clusters.

After successively including the 262 DEGs in univariate Cox regression, LASSO regression, and multivariate Cox regression analysis as candidate prognosis-associated genes, we identified six hub genes (including *CFB*, *IFITM1*, *EPSTI1*, *MARCO*, *CXCL13*, and *CCL5*) from the Ps-score signatures based on their β coefficients (Figures 3E,F, Supplementary Table S8). In addition, the expression of these hub genes was higher in clusterB subgroups. Based on the IHC data from the HPA database, the expression of these hub genes at the protein level was further validated in BC, especially CFB, ESPIT1, and IFITM1 (Figure 3G, Figure 6C). Based on the expression of these genes and their corresponding β coefficients, the Ps-score was defined by the following formula: Ps_score=−0.365×CFB−0.45×IFITM1−0.298×EPSTI1−0.461×MARCO−0.26×CXCL13+0.439×CCL5 (Supplementary Table S9, the gene name represents the corresponding gene expression FPKM values). Subsequently, those patients with TNBC were divided into a high and low Ps-score subgroups with median value (–5.28) as the cutoff; the high Ps-score cohorts exhibited a worse prognosis than that of low Ps-score patients in the TCGA data sets (Figure 4A). To prove the universal value of the Ps-score in TNBC, we also performed survival analysis of this score in four extrinsic GEO cohorts and obtained the same results (Figure 4B).

**FIGURE 5:**
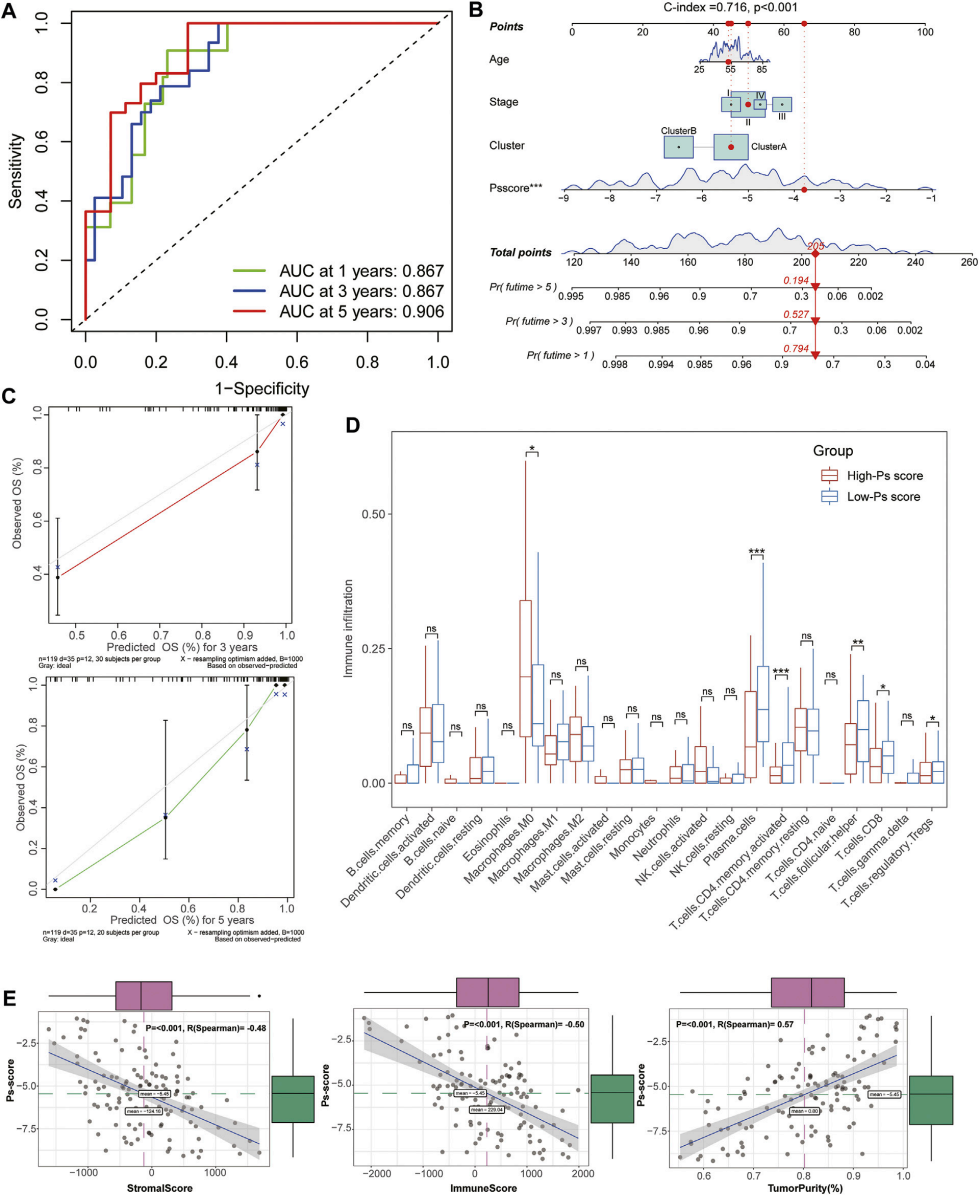
Establishment and evaluation of the Ps-score-related prognostic model for patients with TNBC. **(A)** Time-dependent receiver operating curves of 1-, 3-, and 5-year survival for patients with TNBC using Ps-scores. **(B)** Combined nomogram for predicting the probability of 1-, 3-, and 5-year survival for patients with TNBC. **(C)** Calibration curve of the established nomogram with 3- and 5-year survival, respectively. **(D)** Immune infiltration analysis revealed that substantial immune cells were significantly inhibited in high Ps-score groups. **(E)** Correlation analysis shows the Ps-scores significantly positively associated with the levels of tumor purity (*R* = 0.57) and negatively associated with stromal and immune scores (*R* = –0.48 and *R* = –0.50, respectively).

To investigate the biological characteristics of the Ps-scores, we compared the expression of pyroptosis-related genes and ICI between different Ps-score groups and further explored the correlation between significant clinical phenotypes and the Ps-scores. The results reveal that clusterB possessed a lower level of Ps-scores associated with pharmaceutical and surgical therapy as well as lower pathological stages in patients with TNBC (Figure 4C&E). Interestingly, the expression of pyroptosis-related signatures was significantly increased in the low Ps-score groups, including the CASP, GSDM, and NOD families, as well as inflammatory factors, suggesting the potential role of pyroptosis activation in the low-score of TNBC cohorts with better prognosis (Figure 4D). Moreover, ROC analysis showed 1-, 3-, and 5-year AUC values of the Ps-scores for predicting the prognosis of patients with TNBC of 0.867, 0.867, and 0.906, respectively, in the TCGA sets (Figure 5A). Furthermore, immune infiltration analysis revealed that substantial immune cells were significantly inhibited in the high Ps-score groups, including that of CD8^+^ T cells, follicular helper T cells, activated CD4^+^ memory T cells, and plasma cells (Figure 5D). The correlation analysis also indicates that the Ps-scores were significantly positively associated with the levels of tumor purity (*R* = 0.57, *p* < .001) but negatively associated with stromal and immune scores (*R* = –0.48, *p* < .001 and R = –0.50, *p* < .001, respectively) (Figure 5E).

Based on the Ps-scores and some primary clinical characteristics, multivariate Cox regression analysis was conducted to construct a nomogram that could accurately predict the probability of the 1-, 3-, and 5-year survival for patients with TNBC. The Ps-scores, age, pyroptosis-related cluster, and clinical stages were considered as related predictors for the prognosis of patients with TNBC and incorporated into the nomogram with significant regression coefficients and *p*-values (Figure 5B, Supplementary Table S10). From the nomogram, we could observe that the Ps-score contributed the most to the total score with a 0.716 concordance index (Figure 5B, Supplementary Table S11). Calibration curves exhibited that the nomogram had a good prediction capacity in both 3- and 5-year OS for patients with TNBC (Figure 5C).

### Significance of Ps-Scores in the Prediction of Response to Immunotherapy and Common Chemotherapeutics

**FIGURE 6:**
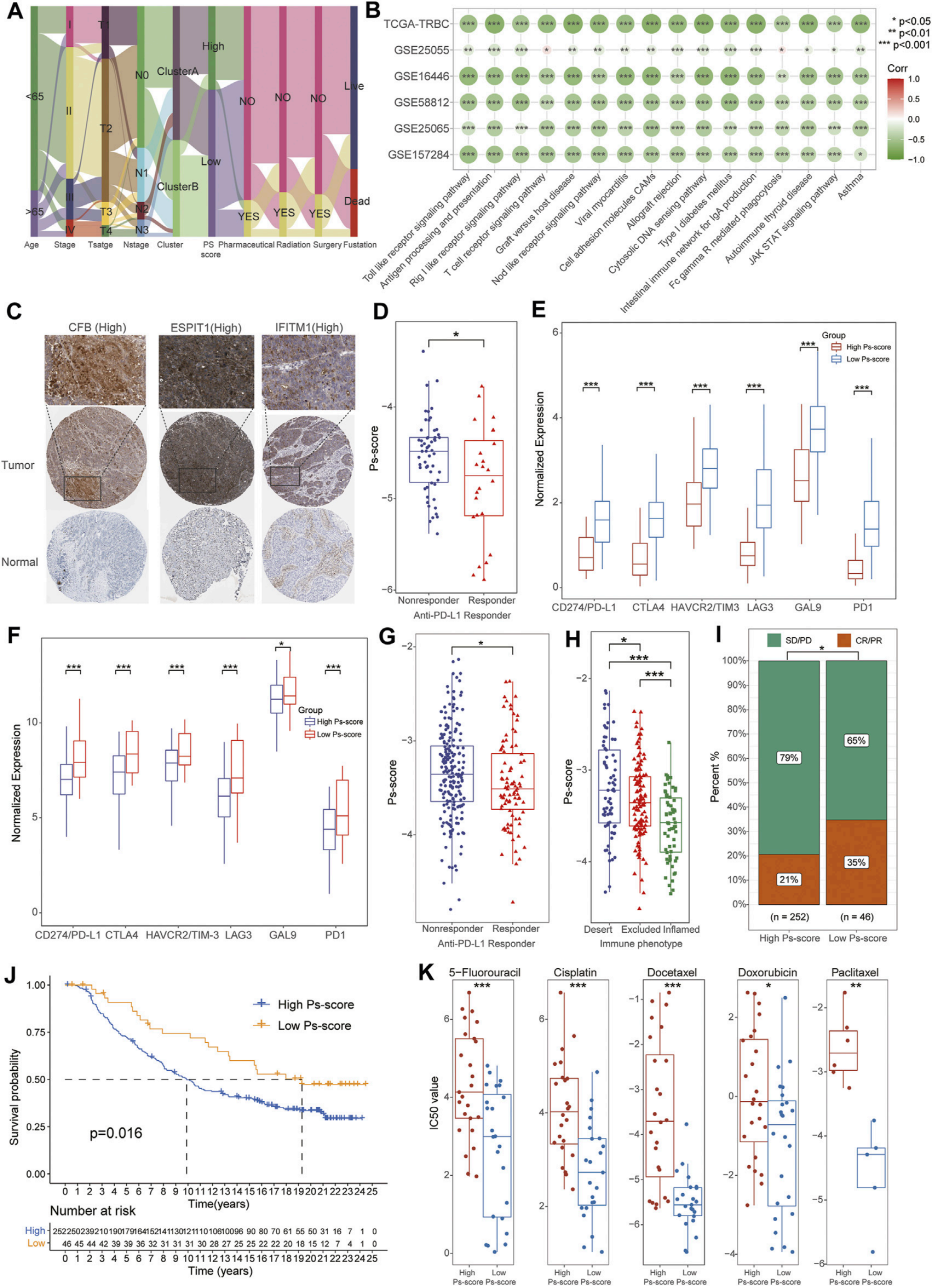
The significance of Ps-scores in the prediction of response to immunotherapy and common chemotherapeutics for TNBC. **(A)** Alluvial diagram visualizing the status changes from different characteristics of patients. **(B)** Correlation analysis of Ps-scores and GSVA pathways in TCGA and GEO data sets. The size of nodes represents the correlation coefficient, and the red or green represents positive or negative correlation, respectively. **(C)** The immunohistochemical results of Ps-score-related genes from the HPA database. **(D)** The box plots displayed patients with better responses to PD-L1 treatment exhibited higher Ps-scores using GSE157284. **(E)** The relative expression of immune-checkpoint genes was significantly increased in the high Ps-scores groups of GSE157284 data sets. **(F)** The relative expression of immune-checkpoint genes was significantly increased in the high Ps-scores groups of IMvigor210 cohorts. **(G)** The box plots indicated patients with better responses to PD-L1 treatment exhibited higher Ps-scores using IMvigor210 cohorts. **(H–I)** Ps-score in different ACRG subtypes and the rate of CR after immunotherapy in IMvigor210 cohorts. **(J)** Kaplan-Meier curves of OS for the patients with TNBC in high and low Ps-score groups of IMvigor210 data sets. **(K)** Comparison of IC_50_ value of 5-fluorouracil, cisplatin, docetaxel, doxorubicin, and paclitaxel in high and low Ps-score groups using GDSC databases.

The alluvial diagram visualized the status changes in the different characteristics of patients (Figure 6A). We found that patients with a low Ps-score in clusterB subtypes had a higher ratio of survival status. When using the TCGA and other external GEO data sets, the results of GSVA demonstrated the coincident negative correlation between Ps-scores and immunoregulation-related pathways, such as the toll-like receptor signaling pathway, antigen processing and presentation, rig I-like receptor signaling pathway, T cell receptor signaling pathway, NOD-like receptor signaling pathway, and JAK-STAT signaling pathway (Figure 6B, Supplementary Table S12).

To further explore the role of Ps-scores in predicting the therapeutic benefit in TNBC, we first calculated the Ps-scores of patients who accepted anti-PD-L1 immunotherapy from the GSE157284 and IMvigor210 cohorts before assigning them into high or low Ps-score groups. From the GSE157284 data set, patients with an effective response rate to anti-PD-L1 therapy showed lower Ps-scores, whereas the relative expression of immune-checkpoint genes was significantly increased in the high Ps-scores groups (Figures 6D,E, Supplementary Table S13). Congruously, we also found that the low Ps-score group had higher expression of immune-checkpoint genes in the IMvigor210 cohort and effective anti-PD-L1 responders also exhibited lower Ps-scores (Figures 6F,G). In metastatic urothelial cancers of the IMvigor210 data sets, distinct immunological subtypes might result in opposite therapeutic responses. Therefore, we also compared the Ps-score levels among these subtypes and found that the lowest Ps-score was in the inflamed subtypes (Figure 6H). Moreover, the rate of complete remission (CR) after immunotherapy was also increased in the low Ps-score cohort compared with the high Ps-score cohort, with the low Ps-score group validated to have a better prognosis for metastatic urothelial cancers (Figures 6I,J). All these results suggested that the Ps-score might serve as a significant indicator in immunotherapy decision making for cancers.

Besides checkpoint blocker therapy, we also investigated the potential associations between the Ps-scores and the curative efficacy of common chemotherapeutics in treating BCs. The IC_50_ value was calculated for five common anti-BC chemotherapeutic drugs obtained from the GDSC databases, including 5-fluorouracil, cisplatin, docetaxel, doxorubicin, and paclitaxel (Supplementary Table S14). Notably, all the drugs exhibited lower IC_50_ values in the low Ps-score groups, indicating patients with low Ps-scores might obtain a better curative efficacy from common chemotherapy (Figure 6K). Collectively, these outcomes indicate that Ps-scores could be associated with the response to immunotherapy and common chemotherapy.

### Validation of Cellular Subtypes in TNBC Through scRNA-Seq Analysis

**FIGURE 7:**
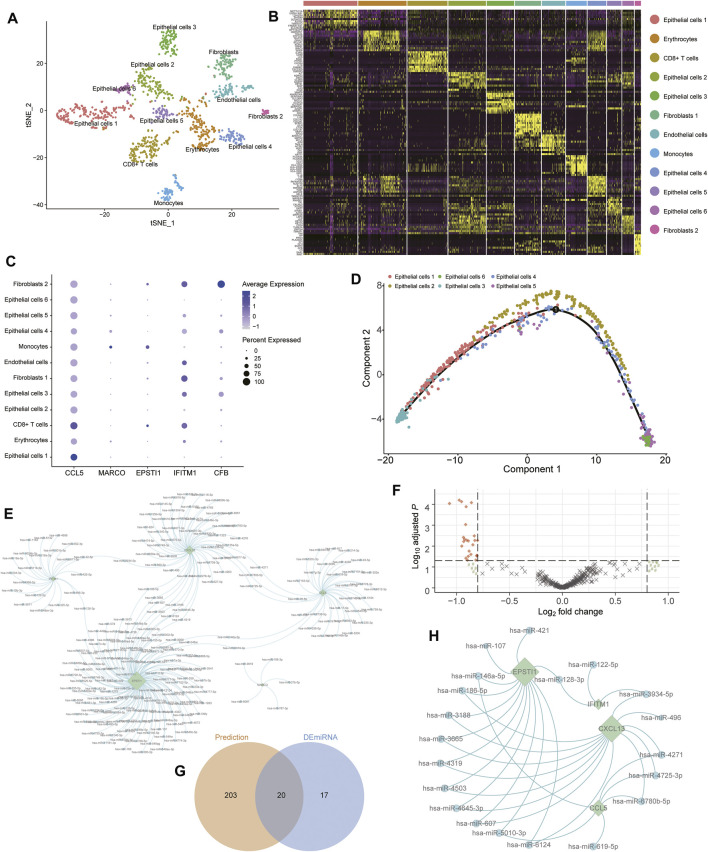
Validation of cellular subtypes in TNBC through scRNA-seq analysis. **(A)** Visualization of tSNE colored according to cell types for TNBC single-cell transcriptomes. **(B)** Heatmap revealing the scaled expression of top 10 marker-genes for each cluster defined in **(A)**. **(C)** Dot plots showing the expression of indicated Ps-score-related genes for each cell cluster. **(D)** Monocle pseudospace trajectory analysis revealing the cellular lineage progression of Epithelial cells subtypes in patients with TNBC colored according to different Epithelial cell clusters. **(E)** The miRNA-hub genes interaction network based on the prediction of four databases. The round node represented the miRNA and the rhomb represented the Ps-score-related genes. **(F)** Volcano plots displayed the upregulated and downregulated DEmiRNAs between two subgroups in patients with TNBC. **(G)** Venn diagram of the predicted miRNA and DEmiRNAs.

To validate the potential subtypes of patients with TNBC, the GSE118389 data set along with 1534 cells from six TNBC tissues were used to identify concrete cellular subtypes and corresponding marker genes. A total of 12 distinct cellular clusters were identified through tSNE analysis (Figure 7A) with the marker-genes of each cluster listed in Supplementary Table S15. Moreover, the results of cell-type annotation using the “SingleR” package indicated these cell clusters fell into six cellular subtypes, including epithelial cells, erythrocytes, CD8^+^ T cells, fibroblasts, endothelial cells, and monocytes, of which the epithelial cells were the most common cell type with six subtypes (Figure 7B). Notably, of the six Ps-score-related genes, five genes were identified as marker genes, and their expression in each cellular subtype is shown in a bubble diagram (Figure 7C). The expression of *CCL5* and *IFITM1* were generally increased in nearly all cellular subtypes and significantly higher than the expression of other signatures. Although the expression of remanent genes was relatively lower in these cells, significant cellular specificity was found in these Ps-score-related genes. For example, *MARCO* was particularly expressed in the epithelial cell subtype 4 and monocytes while *CFB* was particularly expressed in the fibroblast subtype 2 as well as epithelial cell subtypes 3 and 4. Interestingly, *EPSTI1* was significantly expressed in immune-related cellular subtypes including monocytes and CD4^+^ T cells, consistent with the results of IHC. In addition, the pseudotime trajectory analysis also revealed the arrangement of different cellular subtypes that formed a certain rule based on its spatial relationships (Supplementary Figure S2E). Concretely, the trajectory analysis of epithelial cells revealed that a small quantity of epithelial cell subtype 3 was distributed at the start of the trajectory while mixed cells from epithelial cell subtypes 5 and 6 were distributed at the end of the trajectory. Moreover, epithelial cell subtype 2 was uniformly located behind epithelial cell subtypes 1, whereas epithelial cell subtype 4 nearly existed throughout the trajectory (Figure 7D).

### Prediction and Validation of miRNAs Interacted With Hub Ps-Score Genes

To further explore the potential regulatory role of miRNAs targeted to these Ps-score-related genes in TNBC, we found 223 probable miRNAs and successfully constructed the miRNA-hub gene interaction network based on the prediction of the TargetScan, starBase, miRTar, and miRDB databases (Figure 7E, Supplementary Table S16). In addition, to validate the regulatory role of miRNAs in TNBC, we also identified 37 DEmiRNAs between pyroptosis-related clusters, before screening the top 20 miRNAs serving as vital regulatory factors (Figures 7F,G). Finally, we successfully simplified the interaction network with 20 miRNAs and four targeted genes to verify that *ESPIT1* and *CXCL13* are the most active targets regulated by massive DEmiRNAs (Figure 7H).

## Discussion

As a malignant tumor with high mortality, TNBC is known for its poor prognosis, which stems from ineffective therapeutic response to immunotherapy due to tumor biological heterogeneity. Recently, the IMpassion130 trial demonstrated that the combination of atezolizumab, a PD-L1 inhibitor, and nab-paclitaxel could prolong the OS in PD-L1- patients with positive advanced TNBC, heralding the emergence of immunotherapy as an effective treatment strategy for TNBC (Schmid et al., 2018). In addition, The U.S. Food and Drug Administration (FDA) also approved SP142, a PD-L1 IHC assay, as an auxiliary test to identify eligibility for atezolizumab therapy in patients with advanced TNBC. However, the results of IHC staining are still short of high interlaboratory reproducibility with subjective judgment. For example, the IHC levels of PD-L1 were investigated in a total of 443 patients with BC by (Wang et al., 2017) but only ∼16% of these tumors exhibited positive PD-L1 levels. Although the PD-L1 test plays a potentially significant role in the management of multiple advanced carcinomas, objective standardization for this test has not been achieved; hence, its current use in the clinical practice poses a twofold risk to patients: false positive could result in potentially toxic therapies resulting in unforeseen complications, such as miscarriages, or PD-L1 false negatives would benefit from therapy but are excluded from receiving treatment (Reisenbichler et al., 2020). Therefore, identification of novel immunotherapy-related subtyping and reliable objective prognostic indicators for immunotherapy in TNBC is urgently needed.

In contrast to apoptosis, pyroptosis usually occurs in abnormal cells infected by microbes as a positive programmed cell death process, thus inducing the release of pro-inflammatory cytokines and activating an inflammatory response (Bedoui et al., 2020). Prompted by microbes, pyroptosis can also be converted from apoptosis and play various roles in multiple tumors. Pyroptosis has shown antitumor effects through inhibiting the tumor growth in liver and gastric cancers while showing both suppression or promotion effects in BC (Zaki et al., 2010; Chen et al., 2012; Shao et al., 2021). Shi et al. (2015) demonstrated that the activation of the NLRP3 (NOD-, LRR-, and pyrin domain-containing 3) inflammasome was integral for the activation of pyroptosis by recruiting CASP1, further leading to cleavage of GSDMD. In the present study, we explored all the signatures and pathways directly related to pyroptosis in TNBC and detected that the NLRP3/CASP1/GSDMD pathway-related pyroptosis was activated in patients with TNBC, implying that pyroptosis might participate in the mechanism of TNBC, which was associated with the prognosis of TNBC.

The classification of patients based on pathognomonic gene expression profiles is considered a proven method and applied to various studies of TNBC, including autophagy-related signatures (Kim et al., 2012), N6-methyladenosine (Wu et al., 2021), and immune cell infiltration (Harano et al., 2018). In this study, we first proposed a pyroptosis-related molecular subtype based on clustering pyroptosis-related signatures with distinct clinical and immunological characteristics. Interestingly, the characteristics of the two molecular subtypes manifested in significant homogeneity. We detected that patients in ClusterB presented a longer median survival time than those in ClusterA, whereas patients in ClusterB also negatively associated with serious clinical stages, including pathological and TMN stages, suggesting these pyroptosis-related signatures were also significantly associated with different survival risks in patients with TNBC. Our results also reached some consensus: (1) nearly all the pyroptosis-related signatures exhibited higher expression in patients in ClusterB; (2) ClusterB was a specific subtype with a better prognosis and slighter clinical pathological phenotypes; (3) ClusterB was identified as an immune-activated phenotype with higher TME immune scores and infiltration levels of adaptive immune response-related immune cells.

To further explore the potential biological functional features of the pyroptosis-related subtypes in TNBC, we investigated the DEGs between the two subtypes and performed KEGG function enrichment analysis. Consistent with the immunological signatures of subtypes, functional enrichment analysis revealed that immune-activation associated pathways, including natural killer cell-mediated cytotoxicity, the toll-like receptor signaling pathway, chemokine signaling pathway, cytokine-cytokine receptor interaction, NF-kappa B signaling pathway, TNF signaling pathway, and Th17 cell differentiation as well as Th1 and Th2 cell differentiation were significantly enriched in the ClusterB cohorts. Of these pathways, the activation of multiple immune pathways is reported to suppress metastatic spread in TNBC (Zanker et al., 2020) and could be the potential mechanism for a better prognosis of patients in Cluster B.

Furthermore, to increase the clinical application value and create better clinical practicability, we successfully constructed a novel pyroptosis-related scoring tool (Ps-score) to determine the prognostic risk of TNBC based on six hub genes from two clusters. High-expression of these risk signatures at the protein levels was confirmed by IHC from the HPA database, and the Ps-scores effectively stratified patients with TNBC from the TCGA and GEO data set, respectively, into high- and low-risk groups. Survival analysis revealed that the low-score groups had longer OS than patients with high Ps-scores, and ROC curves exhibited a great predictive capacity of Ps-scores for the 1-, 3-, and 5-year survival of TNBC. In addition, the Ps-scores were significantly decreased in the ClusterB cohorts, indicating that the Ps-score could reflect the heterogeneity of patients with TNBC. Moreover, the Ps-score also represented patients with different clinical outcomes and was associated with the response to immunotherapy. The patients with high Ps-scores exhibited worse clinical prognosis and lower expression of immune checkpoints. TME ICI analysis also demonstrated that the Ps-score was significantly negatively correlated with the infiltration levels and tumor purity, suggesting its value in immunotherapy. Finally, a correlation analysis of the Ps-score and pathways based on GSVA results demonstrate a coincident negative relation in both TCGA and other GEO data sets, implying the activation of the various immune-related processes might occur more frequently in low Ps-score cohorts. Notably, besides immune-related pathways activated in low-score patients, massive activation of signal regulatory pathways was observed in high Ps-score groups, including TGF-β1, Wnt, Notch, and the MAPK signaling pathway, which is reportedly involved in the mechanism of TNBC and as target pathways for drug treatments (Giltnane and Balko, 2014; Kim et al., 2016; Pohl et al., 2017; Giuli et al., 2019).

Furthermore, using the IMvigor210 data sets, we also speculated that the Ps-score might be applicable to estimate the clinical response to immunotherapy in other tumors as well. Besides immunotherapy, common chemotherapeutic drugs also demonstrated lower IC_50_ values in the low Ps-score cohorts, including 5-fluorouracil, cisplatin, docetaxel, doxorubicin, and paclitaxel from the GDSC database, implying that these chemotherapeutic drugs would be more effective in patients with TNBC with low Ps-scores. Overall, these findings from external data sets validated the potential benefits of using the Ps-score system and indicated its role in predicting curative responses to common chemotherapies and immune checkpoint therapies.

Finally, the scRNA-seq analysis demonstrated the authentic existence of cellular subtypes with their marker genes in patients with TNBC and clearly showed the distribution of Ps-score-related genes in each subtype. Admittingly, TNBC originated from epithelial cells, and the results of the scRNA-seq also demonstrated multiple subtypes of epithelial cells, reflecting different clusters of tumor cells. Interferon inducible transmembrane 1 (IFITM1) is reported to promote the progression of TNBC through regulating *integrin*, *NFκB*, and *IL6* gene expression and might serve as a novel therapeutic target for patients with *IFITM1* ^+^ TNBC (Provance et al., 2021). Of the Ps-score-related genes, our analysis also detected that *IFITM1* exhibited relatively high expression in epithelial cell subtypes 3–5, consistent with the above patients with *IFITM1* ^+^ TNBC. In previous studies, the epithelial-stromal interaction 1 (EPSTI1) is also shown to modulate the extrinsic apoptotic pathway in TNBC cell lines, which highlighted its potential as a therapeutic target for patients with TNBC (Capdevila-Busquets et al., 2015). Interestingly, *EPSTI1* is overexpressed in monocytes and CD8^+^ T cells, suggesting EPSTI1 might participate in the process of extrinsic apoptosis with the activation of the immune response. Moreover, the pseudotime trajectory analysis displayed the distribution of tumor epithelial subtypes and demonstrated the existence of inner heterogeneity and potential cellular differentiation in patients with TNBC. For the common subtypes of BCs, microRNA profiles from different breast cells were applied to distinguish and reflect different subtypes, including luminal A, luminal B, and basal and malignant myoepithelioma, indicating that the expression of genes in cells could directly reflect the different subtypes in BCs (Bockmeyer et al., 2011). Despite the differences in cellular and individual subtypes, pyroptosis-related signature genes distinguished both subtypes of TNBC based on their differential expression. Combined with the differential expression of miRNAs between pyroptosis-related clusters, we ultimately constructed a miRNA–mRNA interaction network, including 20 miRNAs and four hub genes and found that *EPSTI1* and *CXCL13* were the central nodes with the most miRNA regulation.

Our study has the limitation that the high-throughput sequencing data sets for initial analysis were relatively insufficient as it was simply obtained from public databases. The corresponding results and conclusions remain to be further investigated through more external congeneric research and should be validated via functional experiments *in vivo* and *in vitro*. Furthermore, several conclusions of this study require further research to confirm its reproducibility, improve the clinical application of pyroptosis-related clusters, and elaborate on the role of Ps-scores in predicting the response to immunotherapy for TNBC.

## Conclusion

Our study is the first to propose molecular subtypes based on clustering pyroptosis-related signature expression with distinct clinical and immunological signatures in patients with TNBC. Moreover, we identified and validated a Ps-score system as an effective tool to predict the OS and immunotherapy efficacy in patients with TNBC. Finally, we preliminarily explored the cellular subtypes using scRNA-seq data sets to demonstrate the heterogeneity of TNBC and successfully construct an interaction network to expound the regulatory miRNA targeted Ps-score-related signatures. The various transcriptomic analyses facilitated the screening of significant genetic signatures of TNBC to provide a new clinical application of Ps-scores in predicting prognosis and chemo-immunotherapeutic response for patients with TNBC.

## Data Availability

The original contributions presented in the study are included in the article/Supplementary Material, further inquiries can be directed to the corresponding authors.

## Glossary

AIM2: absent in melanoma 2
BC: breast cancer
CASP1/3/4/5/8: caspase1/3/4/5/8
CDF: cumulative distribution function
CR: complete remission
DEGs: differentially expressed genes
DEmiRNAs: differentially expressed miRNAs
EPSTI1: epithelial-stromal interaction 1
ER: estrogen receptor
FDA: Food and Drug Administration
GDSC: Genomics of Drug Sensitivity in Cancer
GEO: Gene Expression Omnibus
GSDMB: gasdermin B
GSDMD: gasdermin D
GSDME: gasdermin E
GSVA: gene set variation analysis
GTEx: Genotype-Tissue Expression
GZMA: granzyme A
GZMB: granzyme B
HER2: human epidermal growth factor receptor 2
HPA: Human Protein Atlas
IC50: half-maximal inhibitory concentration
ICI: immune cell infiltration
IFITM1: interferon inducible transmembrane 1
IHC: immunohistochemistry
KEGG: Kyoto Encyclopedia of Genes and Genomes
mUC: metastatic urothelial carcinoma
NLR: nucleotide-binding domain and leucine-rich repeat receptor
OS: overall survival
PCA: principal component analysis
PCD: programmed cell death
PD: programmed cell death
PD-L1: PD-ligand 1
PR: progesterone receptor
scRNA-seq: single-cell RNA sequencing
TCGA: The Cancer Genome Atlas
TME: tumor microenvironment
TNBC: triple-negative breast cancer
Tregs: regulatory T cells
tSNE: t-distributed statistical neighbor embedding.
