# Supplementary figures and images for: Characterization of Pyroptosis-Related Subtypes via RNA-Seq and ScRNA-Seq to Predict Chemo-Immunotherapy Response in Triple-Negative Breast Cancer
Source: Front Genet. 2022 Mar 21;13:788670. doi: 10.3389/fgene.2022.788670 (PMC8978671; doi:10.3389/fgene.2022.788670)

A

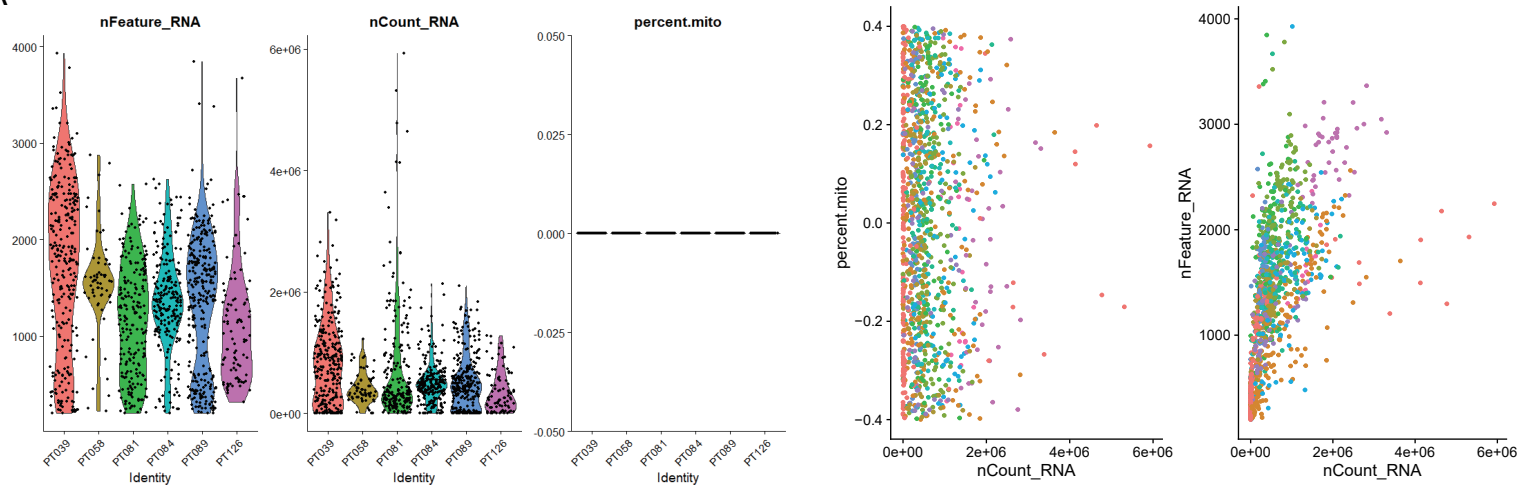

B

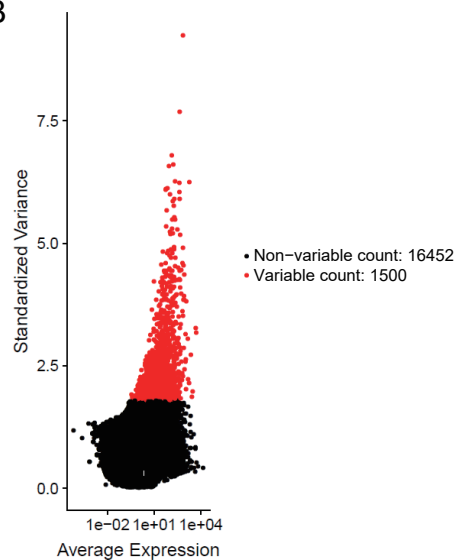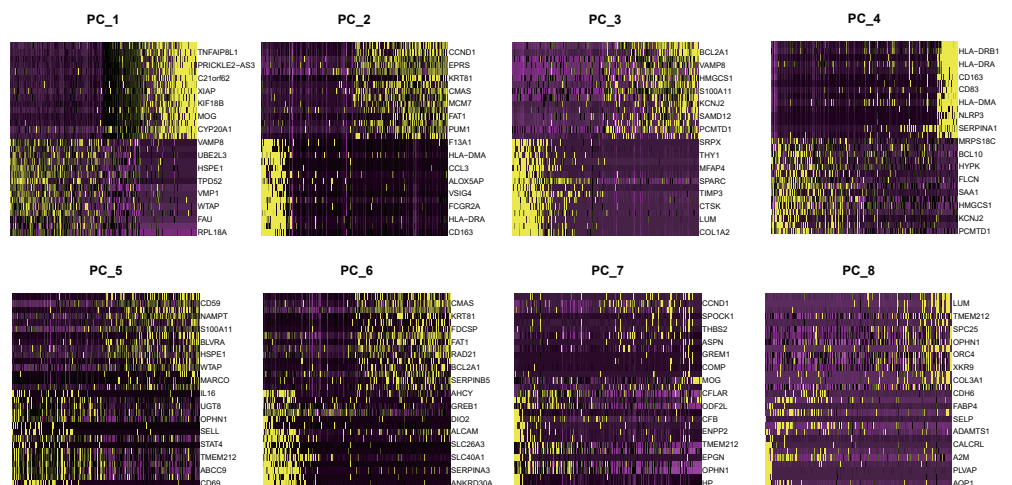

C

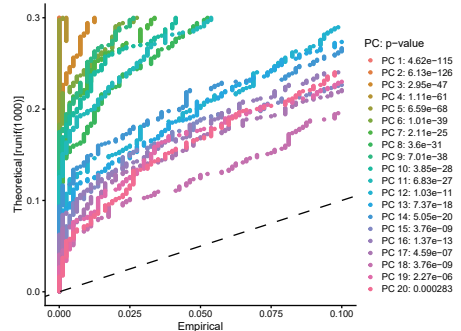

D

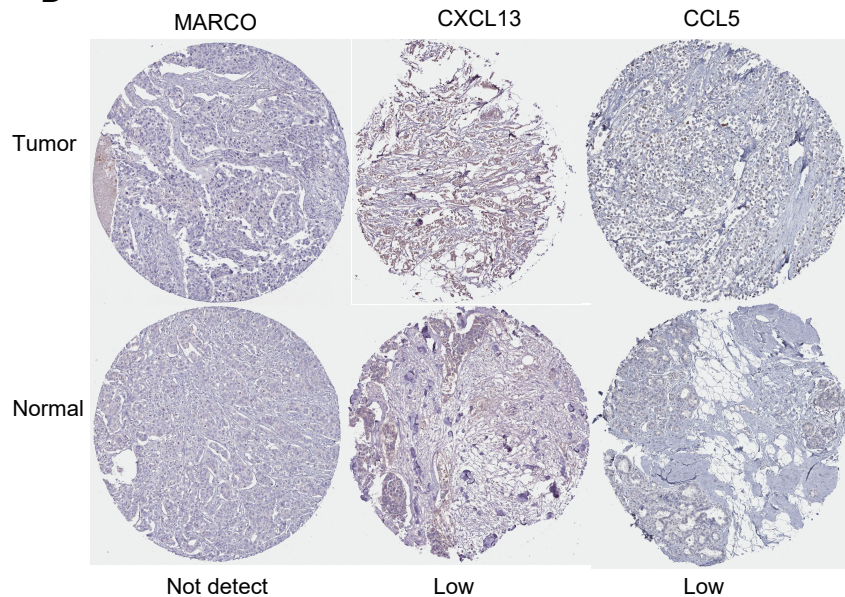

E

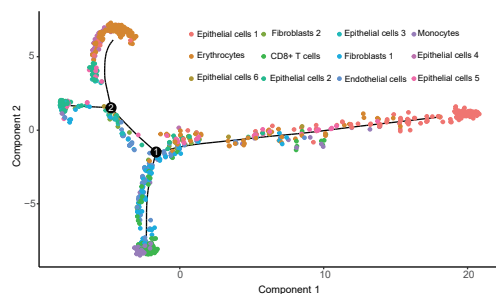

Supplement: Supplementary file 1 [file DataSheet2.PDF]

A

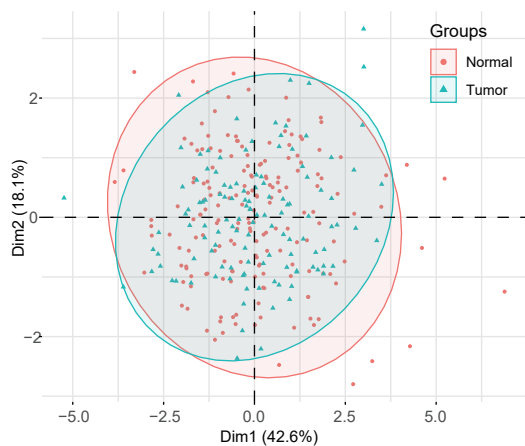

B

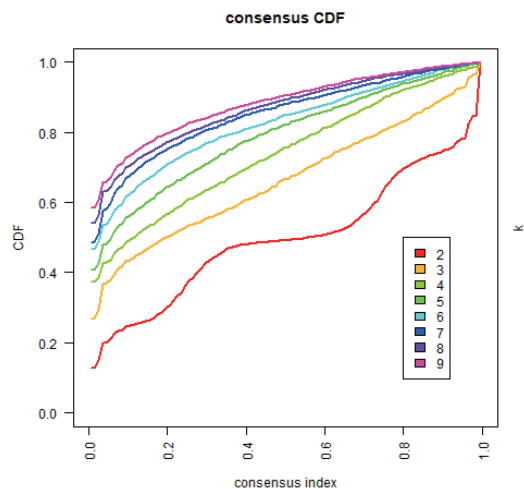

tracking plot

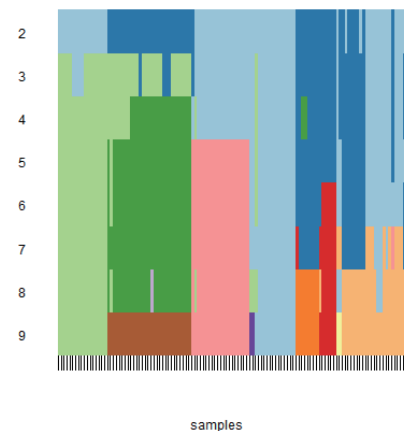

C

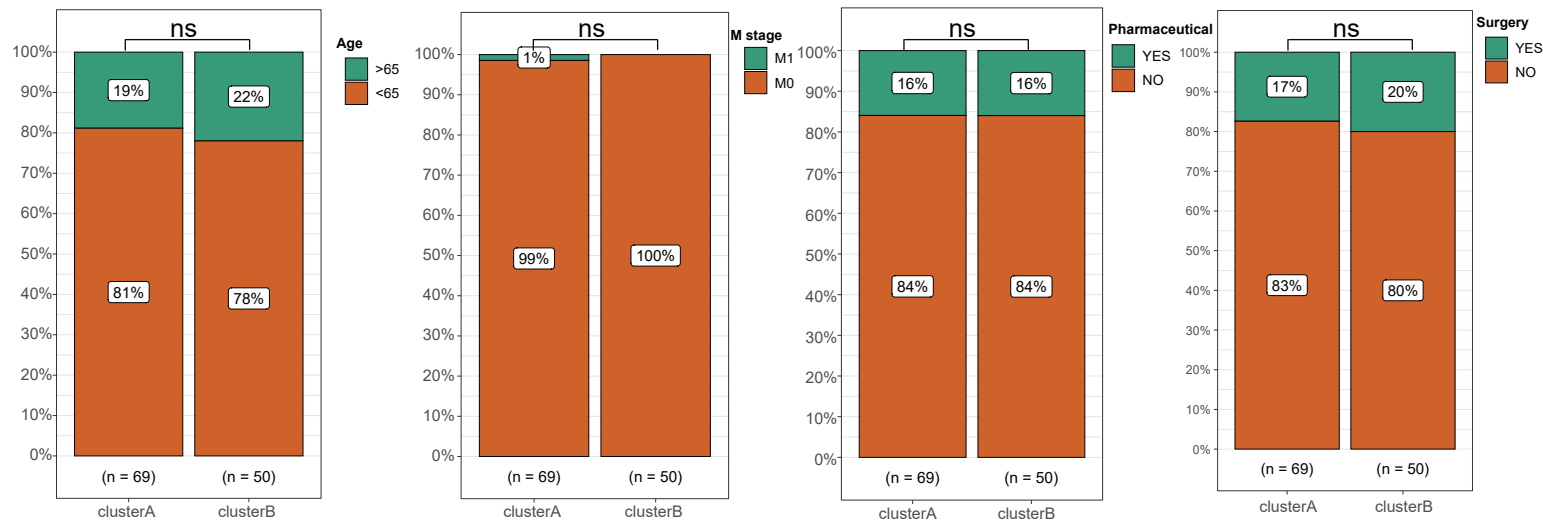

Supplement: Supplementary file 3 [file DataSheet1.PDF]
